# Supplementary material for: circRNA from APP Gene Changes in Alzheimer’s Disease Human Brain
Source: Int J Mol Sci. 2023 Feb 21;24(5):4308. doi: 10.3390/ijms24054308 (PMC10002054; doi:10.3390/ijms24054308)
Supplement: Supplementary file 1 [file ijms-24-04308-s001.zip › ijms-2146764-supplementary-ables circAPP.pdf]

Table S1. Regression coefficients and standard errors of each multivariate linear regression model of each transcript circAPP (hsa_circ_0007556)(A) and *APP* mRNA(B).

A)

Parameter estimates

dependent variable: log(circAPP expression)

Parameter	B	Error Desv.	t	Sig.	Intervalo de confianza al 95%	
					Lower limit	Upper limit
Intersection	-1.535	0.274	-5.595	0.000	-2.090	-0.981
Control	0.307	0.132	2.322	0.025	0.040	0.573
AD	0[b]					
Female	0.034	0.101	0.333	0.741	-0.171	0.239
Male	0[b]					
Age	-0.001	0.003	-0.234	0.816	-0.007	0.006

b. This parameter is set to zero because it is redundant.

B)

Parameter estimates

dependent variable: log(*APP* mRNA expression)

Parameter	B	Error Desv.	t	Sig.	Intervalo de confianza al 95%	
					Lower limit	Upper limit
Intersection	1.993	0.236	8.449	0.000	1.516	2.469
Control	-0.028	0.113	-0.247	0.806	-0.257	0.201
AD	0[b]					
Female	-0.141	0.087	-1.616	0.114	-0.317	0.035
Male	0[b]					
Age	-0.003	0.003	-1.136	0.263	-0.009	0.003

b. This parameter is set to zero because it is redundant.

Table S2. List of KEGG pathways significantly associated with miRNAs predicted to bind circAPP. KEGG pathways were obtained with DIANA mirpath software from the miRNAs predicted with circInteractome.

KEGG pathway	p-value
Wnt signaling pathway	3.32E+05
Long-term potentiation	2.86E+06
Glycosaminoglycan biosynthesis - heparan sulfate / heparin	3.38E+06
Ubiquitin mediated proteolysis	6.68E+06
Axon guidance	7.01E+06
Glutamatergic synapse	7.76E+06
Hippo signaling pathway	1.29E-04
Glioma	1.42E-04
Oxytocin signaling pathway	1.53E-04
Signaling pathways regulating pluripotency of stem cells	2.26E-04
Amphetamine addiction	4.17E-04
Cell adhesion molecules (CAMs)	1.30E-03
TGF-beta signaling pathway	1.80E-03
MAPK signaling pathway	1.80E-03
Pathways in cancer	1.80E-03
Proteoglycans in cancer	1.95E-03
Oocyte meiosis	1.96E-03
Thyroid hormone signaling pathway	2.51E-03
Melanogenesis	2.80E-03
ErbB signaling pathway	3.51E-03
Long-term depression	3.51E-03
Gap junction	4.39E-03
Adrenergic signaling in cardiomyocytes	5.94E-03
Protein processing in endoplasmic reticulum	7.96E-03
Arrhythmogenic right ventricular cardiomyopathy (ARVC)	1.67E-02
Focal adhesion	1.69E-02
Melanoma	1.69E-02
Estrogen signaling pathway	1.69E-02
PI3K-Akt signaling pathway	1.80E-02
Rap1 signaling pathway	1.89E-02
Colorectal cancer	2.37E-02
Mineral absorption	3.52E-02
Cholinergic synapse	3.52E-02
Inflammatory mediator regulation of TRP channels	3.53E-02
Circadian rhythm	3.73E-02
Renal cell carcinoma	3.73E-02
Ras signaling pathway	4.06E-02
Central carbon metabolism in cancer	4.25E-02
mTOR signaling pathway	4.25E-02
cAMP signaling pathway	4.25E-02
Dopaminergic synapse	4.25E-02
Regulation of actin cytoskeleton	4.88E-02

Table S3. Characteristics of the samples included in the study. Nº.: Number; h: hours; PMI: Post-mortem interval.

Nº	Braak stage	ABC score	ABC scale	% β-amyloid plaque area	Gender	Age at death (years)	PMI (h)
1	III	A1B2C1	Low	0.349	Female	96	1.5
2	III	A1B2C1	Low	0.044	Female	88	33
3	I	A1B1C1	Low	0	Female	85	4.3
4	III	NA	NA	0.672	Female	69	14
5	II	A1B1C1	Low	0.147	Female	66	1.4
6	III	A1B2C3	Int	0.648	Female	84	13
7	IV	A2B2C2	Int	0.814	Female	97	NA
8	IV	A2B2C3	Int	0.42	Male	78	5
9	IV	A2B2C2	Int	1.33	Male	84	3.3
10	I	A1B1C1	Low	0	Male	60	15.3
11	V	A3B3C2	High	NA	Male	91	5
12	III	A3B2C3	Int	0.724	Male	83	9
13	IV	A3B2C1	Int	0.131	Female	90	3
14	I	A1B1C1	Low	0.024	Male	85	3.2
15	III	A3B2C3	Int	0.376	Female	85	NA
16	III-IV	A3B2C3	Int	0.357	Female	98	3
17	IV	A2B2C2	Int	0.431	Female	91	10
18	III	A2B2C3	Int	0.528	Female	98	23
19	V	A3B3C3	High	1.142	Female	77	11
20	VI	A3B3C3	High	0.777	Female	86	2.3
21	V	A3B3C2	High	1.61	Female	82	9
22	I	A1B1C1	Low	NA	Female	85	2
23	IV	A2B2C2	Int	0.959	Male	88	3.5
24	VI	A3B3C3	High	2.013	Male	70	2.35
25	VI	A3B3C3	High	1.545	Male	59	4
26	II	A2B1C3	Low	NA	Male	80	3
27	II	A1B1C1	Low	NA	Male	74	2.5
28	II	A1B2C2	Int	NA	Female	71	4
29	II	A2B1C1	Low	NA	Female	80	3.7
30	0	control	Not	NA	Female	43	3
31	0	control	Not	NA	Male	54	18
32	0	control	Not	NA	Female	19	NA
33	0	control	Not	NA	Female	46	7
34	0	control	Not	NA	Male	28	6
35	0	control	Not	NA	Male	41	3.5
36	0	control	Not	NA	Male	54	2.7
37	0	control	Not	NA	Male	81	10.5
38	0	control	Not	NA	Male	26	6.2
39	0	control	Not	NA	Male	53	7
40	0	control	Not	NA	Female	88	9
41	0	control	Not	NA	Male	66	6.5
42	0	control	Not	NA	Female	88	3.5
43	0	control	Not	NA	Female	76	11.5
44	0	control	Not	NA	Male	65	3
45	0	control	Not	NA	Male	83	NA

Table S4. It shows the sequences of the primers used in the different PCRs carried out in this work.

	Forward 5'->3'	Reverse 5'->3'
**circAPP**	CGGTGTTGTCATAGCGACAG	GGCCAAGACGTCATCTGAAT
APP	CAGCCAACACAGAAAACGAAG	TGAGTCATGTCGGAATTCTGC
ACTB	GGACTTCGAGCAAGAGATGG	AGCACTGTGTTGGCGTACAG
GAPDH	ACATCGCTCAGACACCATG	TGTAGTTGAGGTCAATGAAGGG
