# Supplementary material for: circRNA from APP Gene Changes in Alzheimer’s Disease Human Brain
Source: Int J Mol Sci. 2023 Feb 21;24(5):4308. doi: 10.3390/ijms24054308 (PMC10002054; doi:10.3390/ijms24054308)
Supplement: Supplementary file 1 [file ijms-24-04308-s001.zip › ijms-2146764-supplementary-Figures circAPP.pdf]

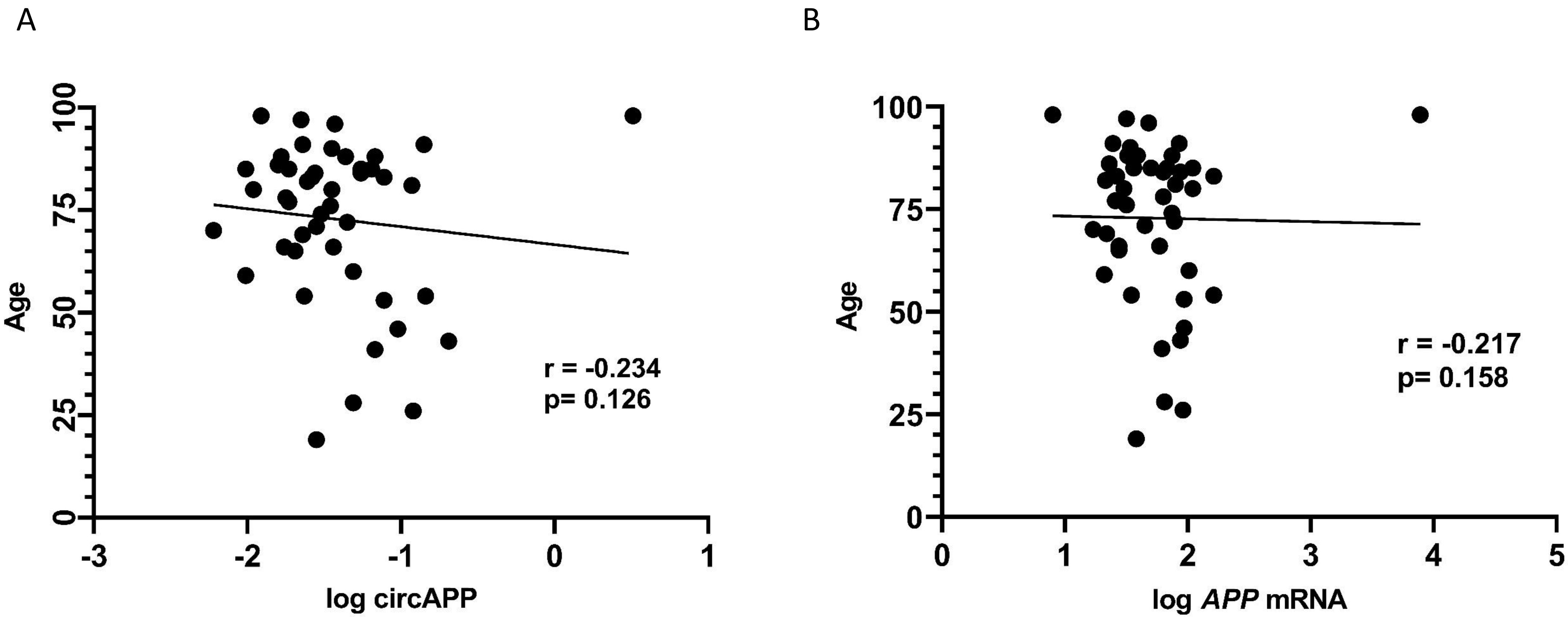

Figure S1. *APP* RNA variants and Age correlation. Dispersion diagram showing no correlation between Age and circAPP (hsa_circ_0007556) and *APP* mRNA expression levels.

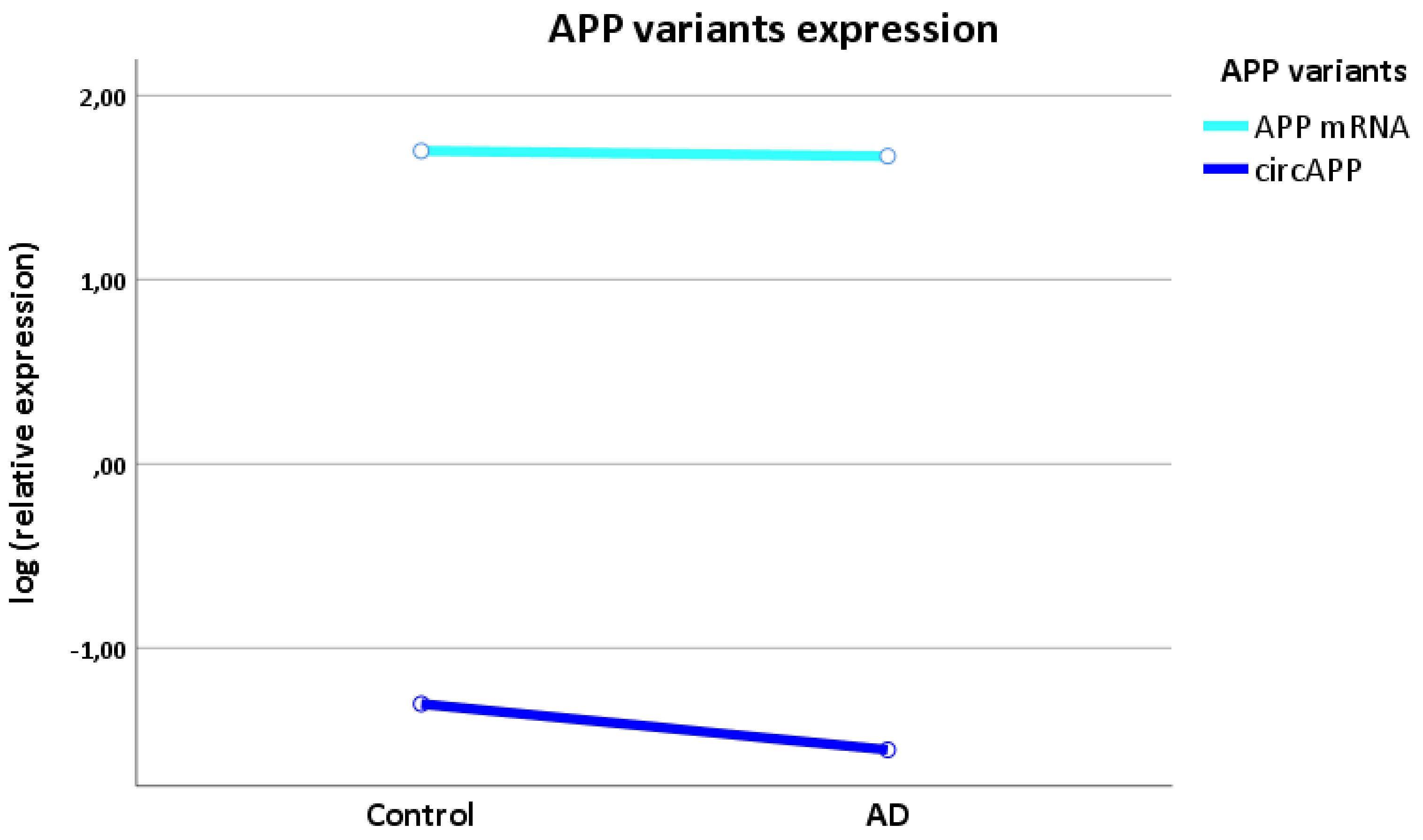

Figure S2. Representation of the proportional expression between controls and AD cases of the log(relative expression) of the two *APP* variants

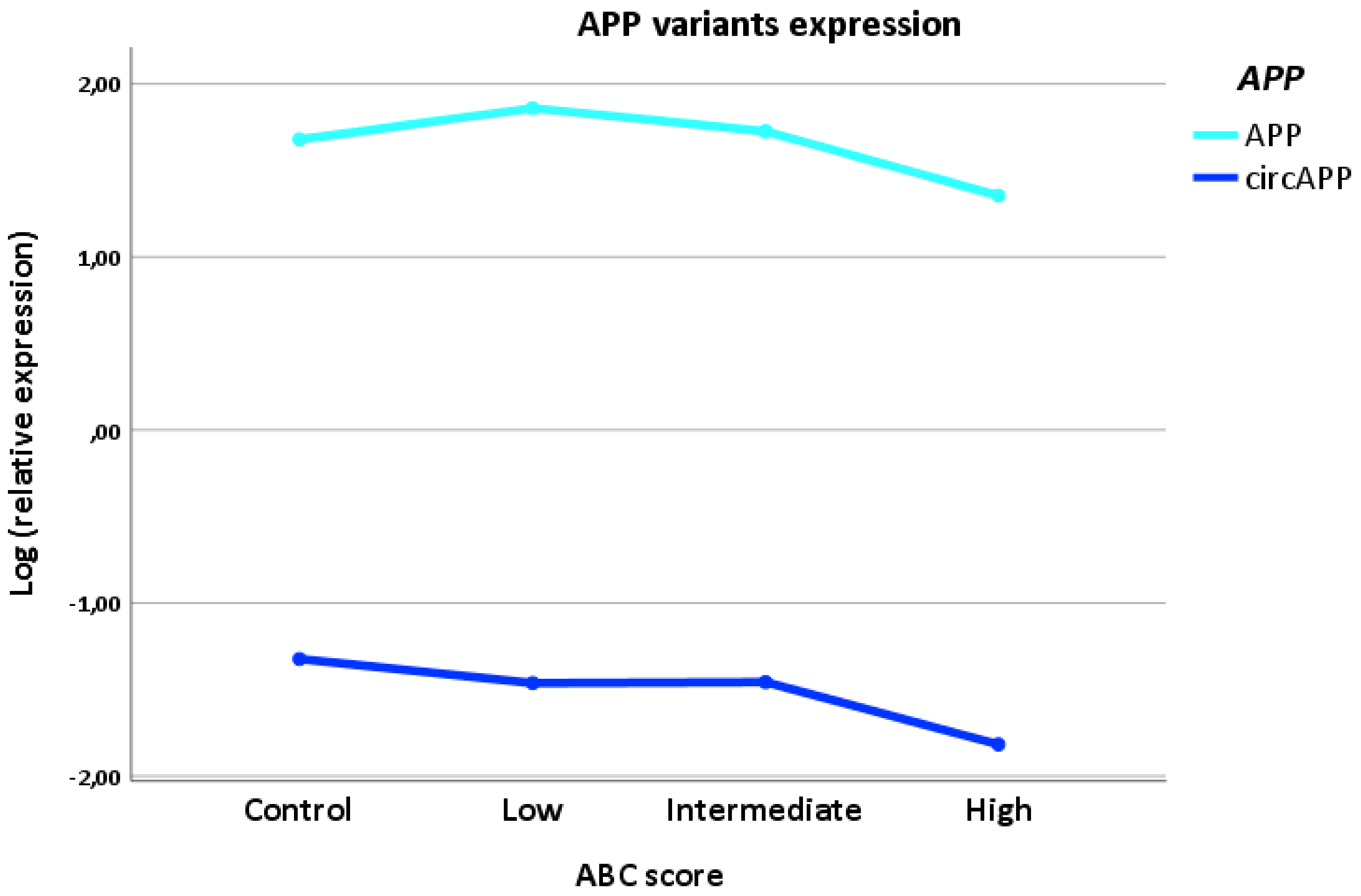

Figure S3. Representation of the proportional expression between controls and AD neuropathological stages, according to the ABC score of the log(relative expression) of the two *APP* variants

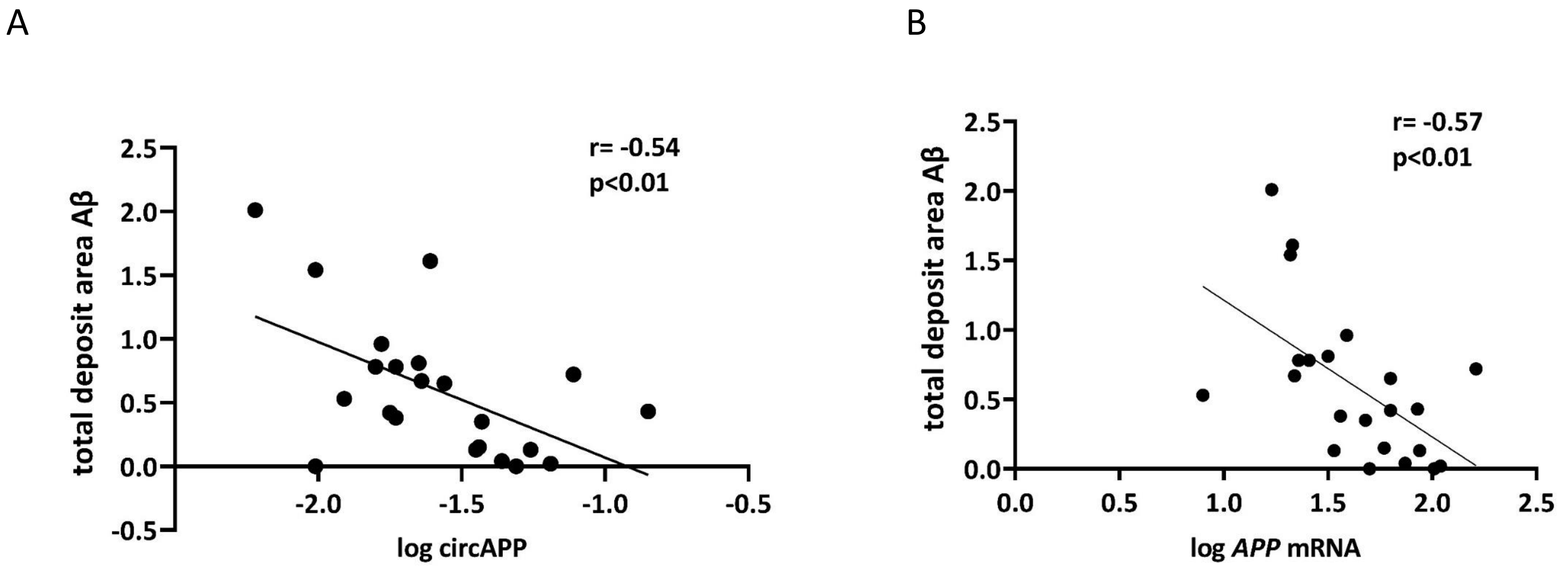

Figure S4. *APP* RNA variants and Aβ deposits correlation. Dispersion diagram showing negative correlation between global average area of Aβ deposits and circAPP (hsa_circ_0007556) and *APP* mRNA expression levels.

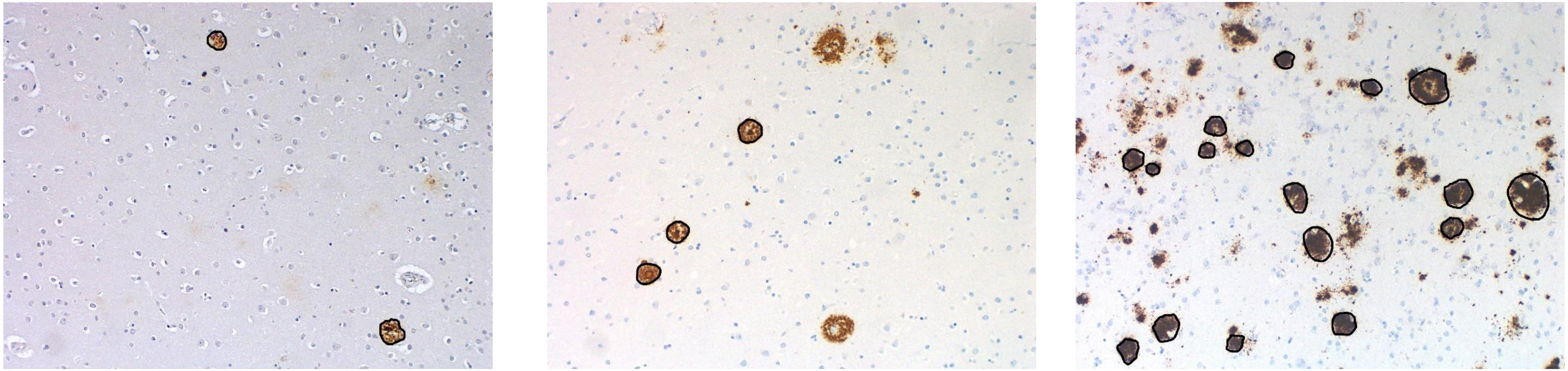

Low Intermediate High

ABC score

Figure S5. Quantitative assessment of Aβ deposits. The images show sections of the entorhinal cortex after immunostaining with a mouse monoclonal anti- Aβ antibody. Aβ plaques are surrounded by a black line. The area enclosed by the black line represents the area of the Aβ plaque measured with the ImagenJ software.
